# Characterization and Histological Examination of a Rare Giant Cell Glioblastoma

**DOI:** 10.7759/cureus.9237

**Published:** 2020-07-17

**Authors:** Sriya V Reddy, Anna E Kaiser, Francis J Liuzzi

**Affiliations:** 1 Medicine, Lake Erie College of Osteopathic Medicine, Bradenton, USA; 2 Anatomy, Lake Erie College of Osteopathic Medicine, Bradenton, USA

**Keywords:** glioblastoma multiforme, giant cell glioblastoma

## Abstract

Glioblastoma multiforme (GBM) is the most common malignant glial cell tumor of the brain. GBM typically occurs in the cerebral hemispheres and is characterized as a grade IV neoplasm due to its highly invasive nature. GBM can be subdivided into two subtypes, gliosarcoma and giant cell (GC) glioblastoma. While there are similarities between the subtypes, the biggest differences are the rate of occurrence with GC accounting for only 1% of cases, and the tendency of GC to occur more commonly in younger aged patients. In this case study, a GC neoplasm is documented in a 68-year-old male cadaver.

## Introduction

Glioblastoma multiforme (GBM) was first characterized in the 1800s and has since been recognized as the most frequent and malignant primary brain neoplasm [1,2]. According to the World Health Organization, GBM is a grade IV diffuse astrocytic tumor and is organized into two subtypes; gliosarcoma and giant cell (GC) glioblastoma [2,3]. GC is a rare form of GBM accounting for only 1% of all brain tumors and 1%-5% of all GBM cases [4,5].

Due to the rarity of its presentation, there is limited knowledge of GC. However, it has been shown that GC and gliosarcoma share several characteristics including a male predominance, similar racial distribution, and comparable tumor size and location [5]. On the contrary, GC tends to present earlier than gliosarcoma, and prognosis, while still poor, is better in patients with GC owing, most likely, to the characteristic histopathologic appearance of the GC cells allowing for more complete resection [5].

GC tumors are characterized by malignant, multinucleated, astrocytic cells [6]. Within the limited research that has been published, the most common reported sites are in the cerebral hemispheres, often in the subcortical substantia alba of the temporal and parietal lobes [7]. Additional reported locations include the frontal lobe, cerebellum, lateral ventricles, optic chiasm, and spinal cord [7,8].

The current case report describes a GC tumor of the right occipital lobe identified in an elderly male cadaver and adds to the exceedingly limited knowledge of this subtype of GBM.

## Case presentation

This case report involves a 68-year-old Caucasian male cadaver who had been diagnosed with GBM. His cause of death was listed as aspiration pneumonia. Specific details of the subject’s clinical history, medical records, and initial presentation have been unobtainable because of the State of Florida Anatomical Board regulations.

**Figure 1:**
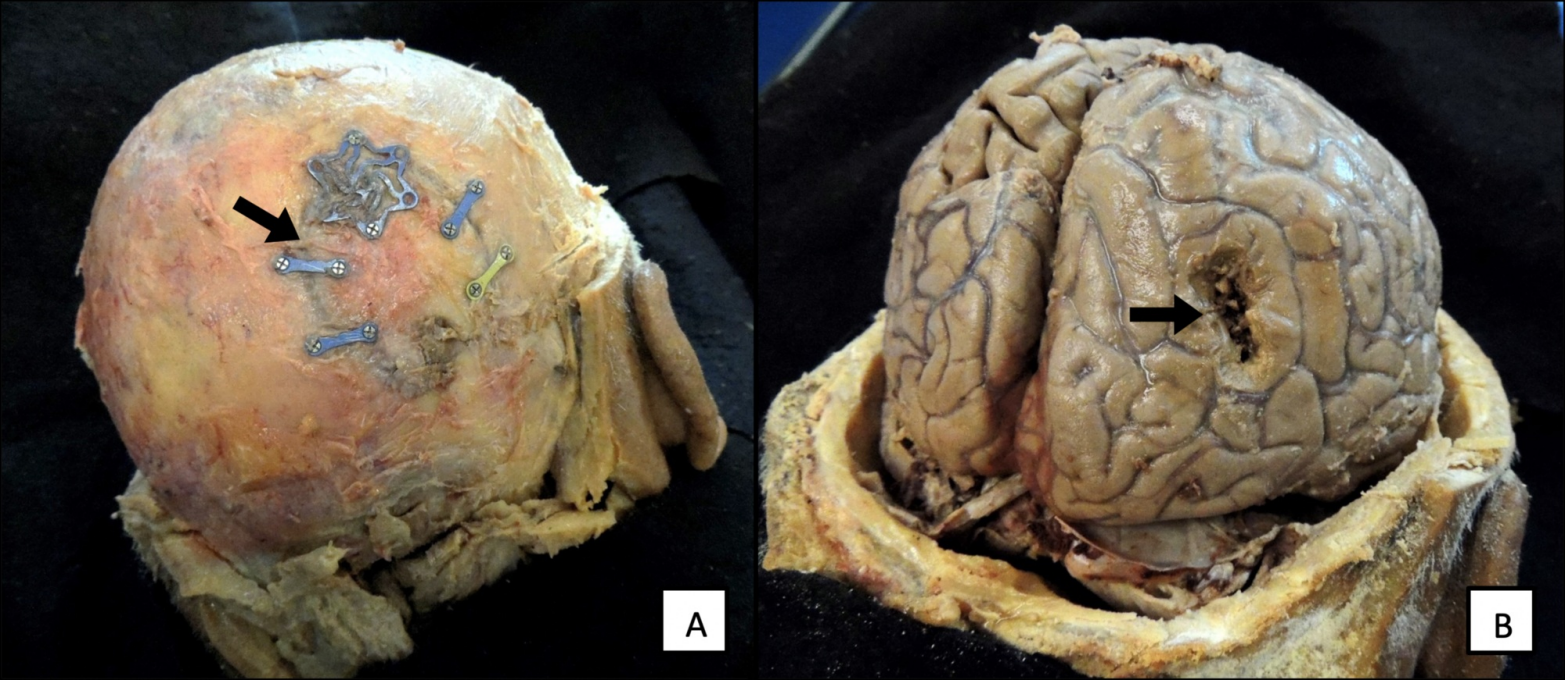
(A) Bone flap from a previous surgical site in a 68-year-old cadaver. (B) Tumor resection site

**Figure 2:**
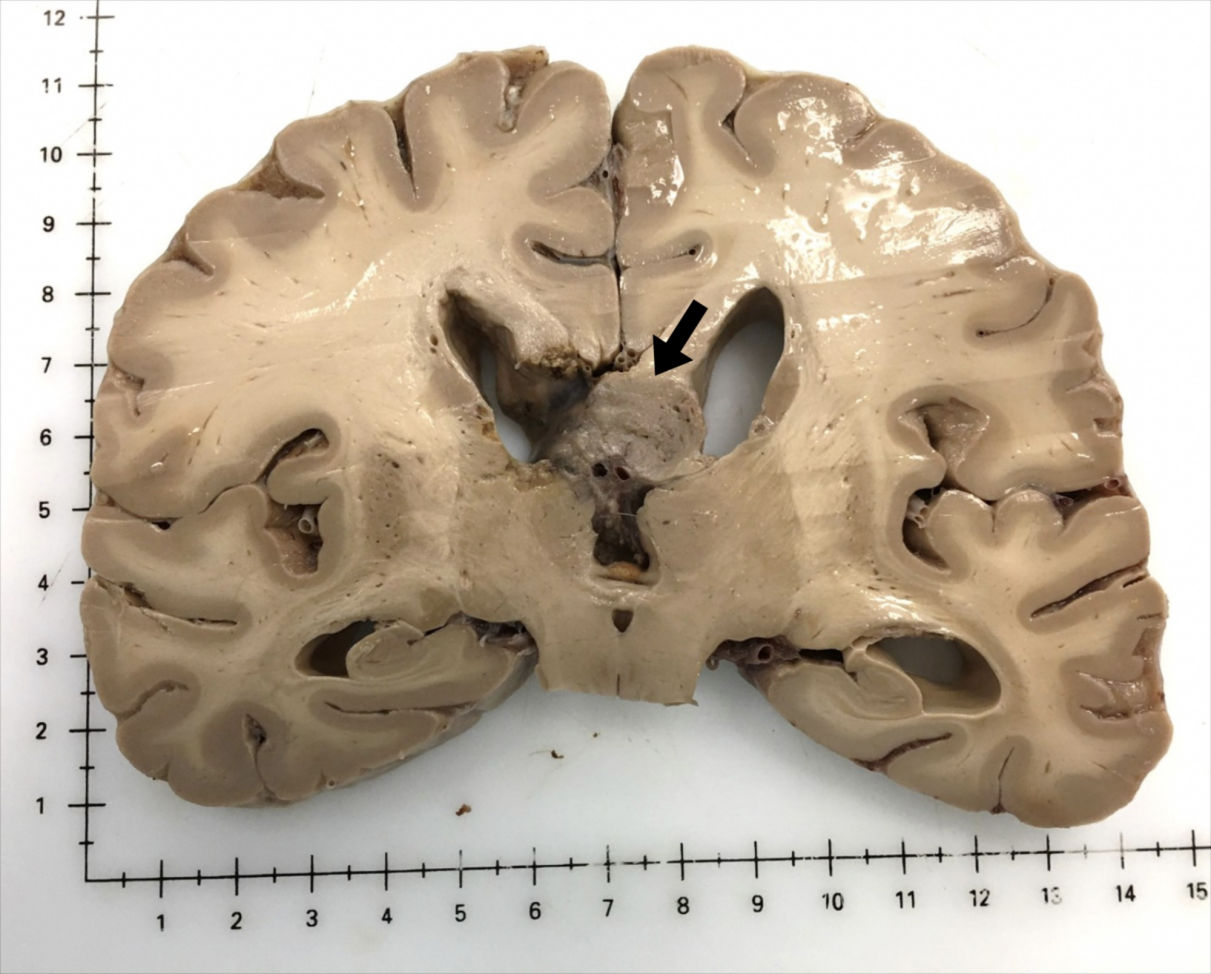
Coronal section showing GBM extension into the lateral ventricles, the body of the fornix, and corpus callosum GBM, glioblastoma multiforme

**Figure 3:**
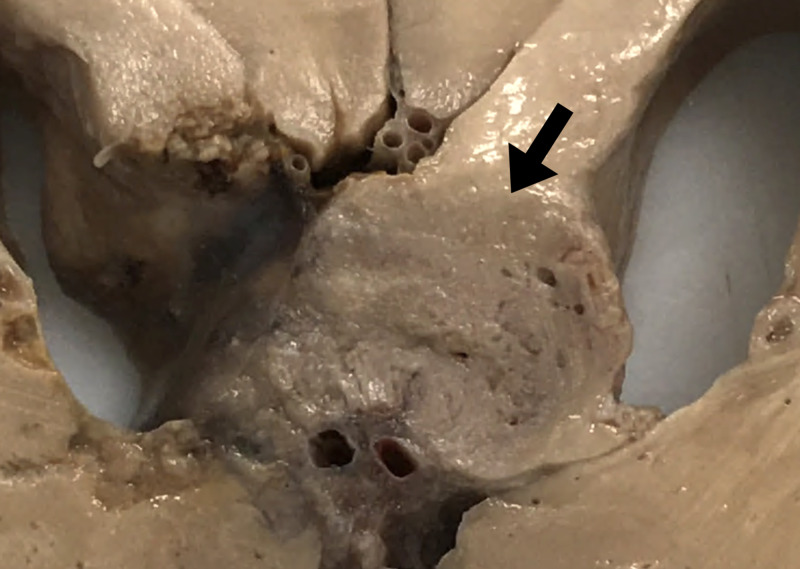
The tumor involves the right corpus callosum and fornix; it extends into the right lateral ventricle as well as across the midline into the region of the left corpus callosum and ventricle

To further investigate the GBM tumor, the scalp was reflected, and a bone flap from a previous surgery was found in the right occipital bone (Figure 1A). Upon removal of the calvaria, it was clear that the supratentorial tumor had been incompletely resected from the right occipital lobe (Figure 1B). Coronal sections of the brain were made, which showed an estimated tumor size of 5.5 cm anterior to posterior and 2 cm in width with extension into the lateral ventricles, the body of the fornix, and corpus callosum (Figure 2). Higher magnification of the tumor showed that it had mostly invaded the right lateral ventricle, but subsequently encroached into the left lateral ventricle region (Figure 3).

**Figure 4:**
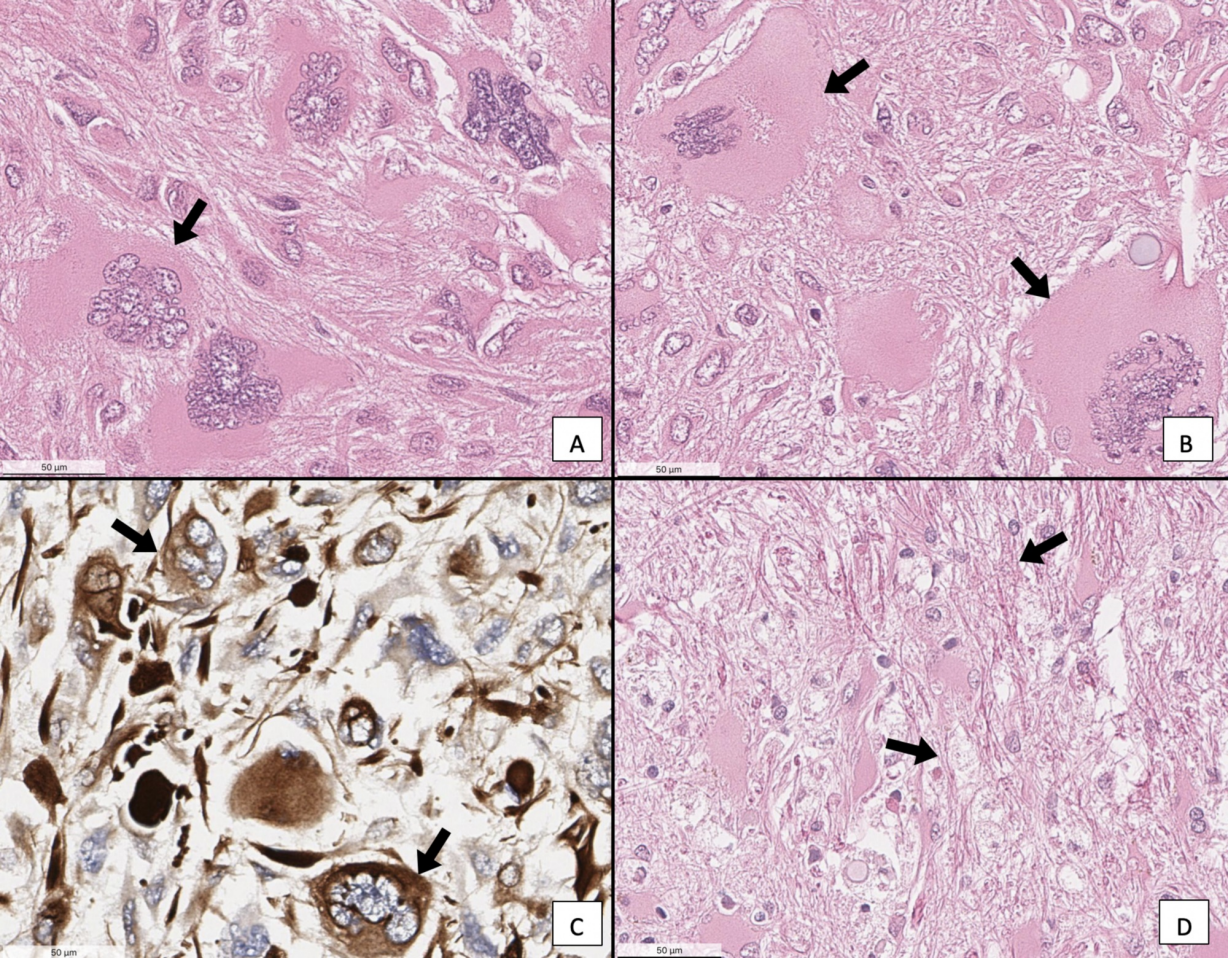
(A&B) H&E stain of GC neoplasm. (C) Vimentin stain of a GC neoplasm. (D) H&E stain of GC neoplasm, showing excess collagen fibers in the extracellular matrix Scale bar: 50 μm. Abbreviations: H&E, hematoxylin and eosin; GC, giant cell

Histopathological examination of the tumor revealed vimentin-positive cells, which indicated the presence of immature astrocytes, characteristic of progressive GBM. Further histological examination of hematoxylin and eosin (H&E)-stained sections revealed that the tumor is a rare subtype of GBM known as GC glioblastoma (Figure 4A, B). Immunohistochemistry analysis to identify markers specific to GC was not performed, however, GC tumors are astrocytic in origin.

The number of multinucleated giant cells per area density was estimated to be 24.6 cells per 0.354375 mm^2^. Of the giant cells, 23 cells per 0.354375 mm^2^ appear to be vimentin-positive (Figure 4C). Further histological analysis revealed intense and abnormal accumulation of collagen fibers throughout the extracellular matrix of the tumor specimen (Figure 4D).

## Discussion

GC, a rare variant of GBM, typically affects Caucasian males between the fourth (21.1 %) and fifth (16.4%) decades of life [5]. The reason for the higher incidence of this neoplasm among the Caucasian population, as well as its root causes, are largely unknown. Researchers report that there is a greater occurrence of GBM in patients with hereditary syndromes such as Turcot syndrome, Li-Fraumeni syndrome, and neurofibromatosis; however, the neoplasm most commonly occurs as a sporadic mutation [9]. Specifically, within the GC subtype, there are higher frequencies of genetic abnormalities than noted in GBM. These include microsatellite instabilities in GC, TP53 mutations, p53 mutations, and deletion of chromosome 10 in all non-giant cells of GC [8].

The histological examination of the GBM from this male cadaver allowed definitive classification as a GC. The abundant eosinophilic cytoplasm, multinucleated cells, and chromatin rich nuclei are all characteristic of GC (Figure 3). Additional studies have identified GC cells as abnormal multinucleated cells characterized by chromatin rich nuclei, prominent nucleoli, and eosinophilic cytoplasm with chromatin deposits [6,7]. While additional immunohistochemistry analysis was unable to be performed, these cells did stain positively with vimentin (Figure 3C). Vimentin is part of the type III intermediate filament family located within the cytoplasm of mesenchymal cells, and its overexpression plays a role in GBM cell invasion and proliferation [10]. Previous studies have shown that GC cells are known to be vimentin-positive as well as glial fibrillary acidic protein, S100 protein, and alpha-1 antichymotrypsin positive [7,8]. It was clear after histological examination that this individual had suffered from the rare GC subtype of GBM.

The most common locations for GBM are in the frontal and temporal lobes with only 4.2% of GBM occurring in the occipital lobe [5,8]. Kozak and Moody reported that of the 1% of GBM that were classified as GC, 4.1% were found in the occipital lobe [5]. Additionally, Jin and colleagues reported that out of 683 patients, only 2.5% of GC cases were found in the occipital lobe compared to 4.3% of all GBM cases; showing that this case report details an extremely rare glial cell tumor presentation [11].

Patients with tumors occupying the occipital lobe typically present with headache, visual field deficits, visual hallucinations, and prosopagnosia, which is the inability to recognize familiar faces [12]. Moreover, patients suffering from GBM commonly present with progressive hemiparesis, headache, nausea, and vomiting [13]. Specifically, in patients with tumors invading the corpus callosum, known as butterfly tumors, common clinical presentations are progressive headaches and hemiparesis [14]. In the male cadaver presented in this case report, the primary tumor likely originated in the right occipital lobe with subsequent anteromedial growth of the tumor into deeper brain structures such as the corpus callosum.

Based on the size of the lesion in the occipital lobe, it is apparent that the aim of the surgery was to debulk the GC neoplasm. It is not known whether this individual received additional treatment such as radiation or chemotherapy. While the GC was not the reported cause of death of this individual, it certainly would have presented with worsening signs and symptoms, which would ultimately lead to death.

Treatment and prognosis reports for GC have only recently been published. Between the years of 2010 and 2014, the majority (69.7%) of GC patients who underwent biopsy or surgery also received radiation therapy, and of those radiation therapy patients, 90.3% also received adjuvant chemotherapy [11]. Additionally, patients who received both radiation and chemotherapy after biopsy and/or surgery, known as trimodal therapy, showed significantly increased survival (17.55 months) compared to those who did not receive additional treatments (6.68 months) [11]. The results from Jin and colleagues support previous research that stated that GC patients tend to have longer survival compared with other GBM histologies as well as indicate that trimodal therapy lends to extended survival [5,11]. Finally, their work identified younger age, higher income, and being female as positive prognostic factors for patients diagnosed with GC [11]. From these results, it is clear that early identification of a GBM leads to a better prognosis, and that the GC subtype provides favorable outcomes in comparison to gliosarcoma. Lastly, trimodal therapy is the most aggressive treatment option, but it provides the greatest survival time among GBM patients, including GC patients.

## Conclusions

GBM is the most common malignant brain tumor in adults with a late-onset and poor prognosis. On the contrary, GC is a rare subtype of GBM that not only presents at a younger age but also has a slightly increased survival rate. The primary GC tumor most commonly originates in the frontal and temporal lobes. This case report presents a 68-year-old male who was diagnosed with GC that most likely originated in the right occipital lobe and spread medially into the corpus callosum. Histological analysis of the tumor revealed numerous vimentin-positive giant cells per area density, with an abnormally high accumulation of collagen fibers and connective tissue deposits. Due to the limited number of case reports on GC, it is necessary to present new findings to add to the current knowledge base and better understand GC tumor behavior. Though the extent of treatment for this particular patient is unknown, those who receive trimodal therapy, including safe surgical resection of the tumor, radiation, and chemotherapy, seem to have a favorable prognosis.
